# Humoral Response of Buffaloes to a Recombinant Vaccine against Botulism Serotypes C and D

**DOI:** 10.3390/toxins9100297

**Published:** 2017-09-22

**Authors:** Denis Y. Otaka, José D. Barbosa, Clóvis Moreira, Marcos R. A. Ferreira, Carlos E. P. Cunha, Antônio R. S. Brito, Rafael A. Donassolo, Ângela N. Moreira, Fabrício R. Conceição, Felipe M. Salvarani

**Affiliations:** 1 Instituto de Medicina Veterinária, Universidade Federal do Pará, BR 316 Km 61, Saudade II, Cristo Redentor, Castanhal 68740-910, PA, Brazil; otaka@veterinario.med.br (D.Y.O); diomedes@ufpa.br (J.D.B.); silva.rod1996@gmail.com (A.R.S.B); 2 Centro de Desenvolvimento Tecnológico, Núcleo de Biotecnologia, Universidade Federal de Pelotas, Pelotas 96160-000, RS, Brazil; clovismoreirajr@live.com (C.M.J.); marcosferreiravet@gmail.com (M.R.A.F.); cpouey@gmail.com (C.E.P.C.); rafaeldonassolo@hotmail.com (R.A.D.); angelanmoreira@yahoo.com.br (Â.N.M.); fabricio.rochedo@ufpel.edu.br (F.R.C.)

**Keywords:** *Clostridium botulinum*, botulinum neurotoxins, BoNT, antitoxins

## Abstract

Botulism is a fatal intoxication caused by botulinum neurotoxins (BoNTs), which are mainly produced by *Clostridium botulinum* and characterized by flaccid paralysis. The BoNTs C and D are the main serotypes responsible for botulism in animals, including buffaloes. Botulism is one of the leading causes of death in adult ruminants in Brazil due to the high mortality rates, even though botulism in buffaloes is poorly reported and does not reflect the real economic impact of this disease in Brazilian herds. Vaccination is reported as the most important prophylactic measure for botulism control, although there are no specific vaccines commercially available for buffaloes in Brazil. This study aimed to evaluate the humoral immune response of buffalo groups vaccinated with three different concentrations of recombinant proteins (100, 200, and 400 µg) against BoNTs serotypes C and D as well as to compare the groups to each other and with a group vaccinated with a bivalent commercial toxoid. The recombinant vaccine with a concentration of 400 μg of proteins induced the highest titers among the tested vaccines and was proven to be the best choice among the formulations evaluated and should be considered as a potential vaccine against botulism in buffalo.

## 1. Introduction

Botulism is a fatal intoxication caused by botulinum neurotoxins (BoNTs) and characterized by flaccid paralysis due to the inhibition of acetylcholine release at the neuromuscular junction. BoNTs are mainly produced by *Clostridium botulinum*, which is an obligatory anaerobic Gram-positive bacillus that is ubiquitous in nature and are capable of growing in decaying organic matter. However, under adverse environmental conditions, *Clostridium botulinum* can survive for long periods in its spore form [1,2,3,4]. There are seven types of BoNTs (A–G) classified according to their antigenicity, although they all have similar mechanisms of action [1,2,3]. The BoNTs C and D are the main serotypes responsible for botulism in animals, including buffaloes [5,6]. Botulism is one of the leading causes of death in adult ruminants in Brazil due to the high mortality rates [7], even though botulism in buffaloes is poorly reported [5,6,8] and does not reflect the real economic impact of this disease in Brazilian herds. Vaccination is reported as the most important prophylactic measure for botulism control [1,4]. However, there are no specific vaccines commercially available for buffaloes in Brazil, so buffalo breeders use bovine vaccines in their herds with controversial induction of protective antibodies. Commercially available vaccines against botulism are based on toxoids made from BoNTs purified from *C. botulinum* cultures. However, this method of production has some limitations: it involves a high risk as BoNTs are the most potent biological toxin known and it also leads to an unpredictable production since this process yields variable amounts of BoNTs between batches [1]. Recombinant DNA technology is an alternative methodology to vaccine production, which can overcome these limitations as it can be produced efficiently in large amounts and result in no toxicity [1,3,4]. Therefore, some studies involving the production and evaluation of recombinant vaccines against animal botulism have been reported [1,3,4], but none of them evaluated the humoral immune response of buffaloes to a recombinant vaccine against botulism serotypes C and D.

This study aimed to evaluate the humoral immune response of buffaloes 56 days after vaccination at different concentrations (100, 200, and 400 µg) of recombinant proteins (Vrec) against BoNTs serotypes C and D as well as to compare it with a bivalent commercial toxoid (Vcom). The recombinant vaccine with 400 µg of recombinant proteins proved to be the best choice among the evaluated vaccines.

## 2. Results

No adverse reactions were observed in the animals inoculated with the recombinant vaccines as expected since the recombinant proteins are non-toxic. This also indicates the innocuity of the vaccine formulation. Animals inoculated with saline did not develop detectable titers against BoNTs C nor D as expected. Animals vaccinated with the different formulations developed different levels of the protective immune response against BoNTs C and D. The minimum level of neutralizing antibodies requested by Brazilian Ministry of Agriculture Livestock and Food Supply (MAPA) are 5 IU/mL and 2 IU/mL for BoNTs C and D [9], respectively. Only Vrec200 and Vrec400 could induce an immune humoral response above the minimum titers required by Brazilian legislation in 100% of the animals vaccinated for both BoNTs. Vrec100 achieved seroconversion rates of 50% and 70%, while Vcom had seroconversion rates of 90% and 80% for serotypes C and D, respectively. The results are shown in Table 1.

Animals vaccinated with Vrec400 obtained the higher antibody titers for both BoNTs C and D. Animals vaccinated with Vrec200 had higher titers than those vaccinated with Vrec100. However, there were no statistical differences between titers of animals vaccinated with Vrec200 and Vcom or Vrec100 and Vcom (*p* < 0.001) (Table 2).

**Figure 1:**
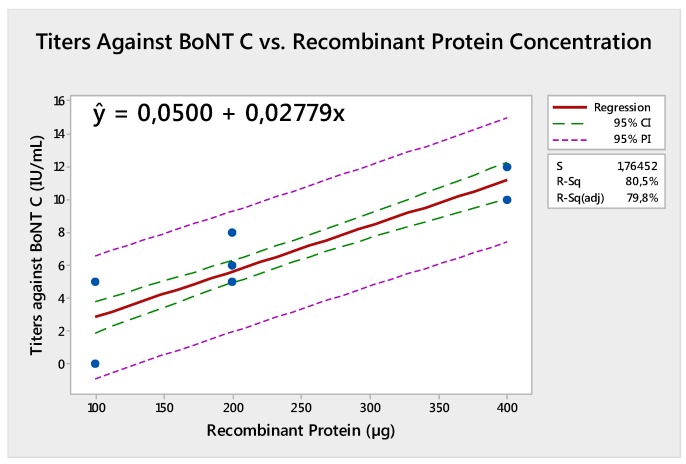
Equation of regression for titers against botulinum neurotoxin (BoNT) C compared to recombinant protein concentration.

**Figure 2:**
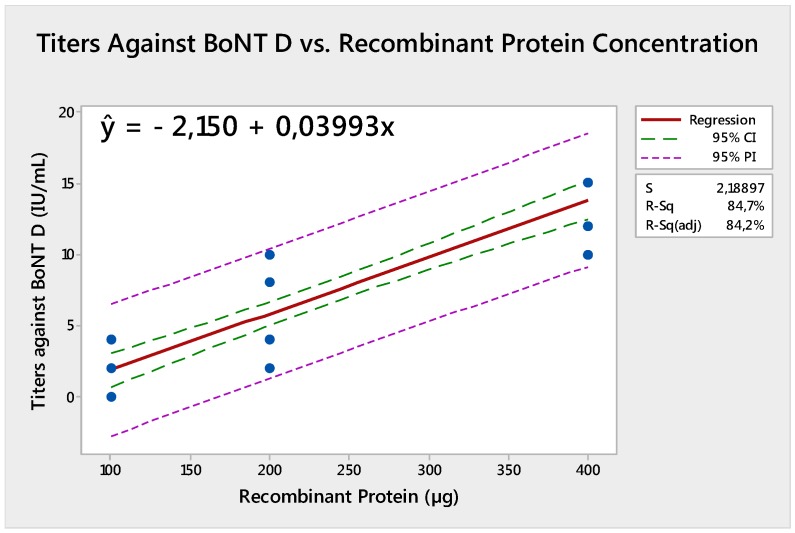
Equation of regression for titers against botulinum neurotoxin (BoNT) D compared to recombinant protein concentration.

The results showed a strong increasing linear relationship between the recombinant protein concentration and titers against BoNT C (r^C^ = 0.897) (*p* < 0.001) in addition to the recombinant protein concentration and titers against BoNT D (r^D^ = 0.92) (*p* < 0.001). Regression analysis was significant and relevant equations are presented in Figure 1 and Figure 2.

## 3. Discussion and Conclusions

According to the Food and Agriculture Organization of the United Nations, the population of buffaloes (*Bubalus bubalis*) worldwide is approximately 195 million and Brazil is the largest producer of buffaloes in the Western world with a herd of approximately 1.3 million animals [10]. Most of the Brazilian animals (493,000, 37.4%) are located in the state of Pará [11], where they are reared within the Amazon biome and are very well adapted to the tropical climate and created mainly in extensive system [12]. This breeding system increases the exposure of these animals to health hazards and recently, our research group reported the occurrence of three outbreaks of botulism in buffaloes that affected two properties in the state of Pará [5].

To our knowledge, this is the first immunological evaluation in buffaloes vaccinated against botulism. In 2013, Gil et al. [1] immunized guinea pigs with a purified recombinant chimera (200 µg) and induced up to 5 and 10 IU/mL of antitoxins against BoNTs serotypes C and D, respectively. Using the same formulation, Cunha et al. [3] immunized cattle and achieved 5 and 6 IU/mL of antibodies against BoNTs C and D, respectively. This study was conducted using the same non-purified recombinant vaccine formulation that Moreira et al. [4] used to immunize guinea pigs. They induced up to 13 and 21 IU/mL of antitoxins against BoNTs C and D. Although that study [4] used a single concentration of 200 µg of recombinant proteins, the present study used three different concentrations: 100, 200, and 400 µg. These concentrations induced mean titers of 2.5 and 1.6; 6.1 and 6.2; and 11.0 and 13.7 against BoNTs C and D, respectively. It can be observed that even the formulation with a higher concentration (Vrec400) was unable to induce serological titers in buffaloes close to the values described by Moreira et al. [4] in guinea pigs. These results can be justified due to the immunological differences between the species and the significant difference in body weight of the animals since buffaloes are much larger animals. Due to this significant difference in body weight, the concentration of the vaccine in the circulation is much lower than that of guinea pigs with the same amount of injected recombinant protein. These considerations reinforce the importance of conducting bioassays in the target species. However, it should be noted that the serological titers induced by Vrec400 (11 IU/mL for BoNT/C and 13.7 IU/mL for BoNT/D) were more than double that of BoNT C and more than six times that of BoNT D for the minimum titers required by NI 23 [9]. Titers of both BoNT C and D were more than double that reported by Cunha et al. [3], who used a bioassay with recombinant chimera inoculated in cattle. The results of this study raise the possibility of considering future studies with a single dose of vaccine at concentrations of 200 µg or 400 µg or two vaccinations with lower concentrations of recombinant proteins. According to the regression equations, only about 180 µg of recombinant proteins of BoNT C and nearly 105 µg of recombinant proteins of BoNT D are needed to stimulate the humoral immune response in order to satisfy the Brazilian legislation in approximately 80% of animals vaccinated. This can be considered a satisfactory immune response in a herd level. Despite the existence of a law that stipulates minimum titers for the approval of a vaccine against botulism in Brazil, it is necessary to emphasize that the clinical picture of botulism is dependent on the amount of toxin ingested. To the veterinary practitioner, it is difficult to know the amount of BoNT ingested by the animal and therefore, it is desirable that the vaccines used should be able to induce the highest degree of immunity for the longest period possible. In conclusion, the recombinant vaccine with a concentration of 400 μg of proteins attained the minimum titers of antibodies required by Brazilian legislation and induced the highest titers among the tested vaccines. Thus, it was proven to be the best choice among the formulations evaluated and should be considered as a potential vaccine against botulism in buffalo.

## 4. Materials and Methods

### 4.1. Ethics Statement

This study was conducted according to the Brazilian National Council for Animal Experimentations (CONCEA) and was approved by the Federal University of Pará Ethics Committee on the Use of Animals (CEUA/UFPA) permit number 9668220616.

### 4.2. Vaccines

A recombinant vaccine containing non-purified BoNTs serotypes C and D was prepared as described by Moreira et al. [4]. After being approved in a sterility test, three different concentrations of recombinant proteins (100, 200, and 400 µg for each serotype) were used, although there was an equal final volume of 5 mL per dose. A commercial bivalent botulinum vaccine containing C and D toxoids was also used.

### 4.3. Vaccination of Buffaloes

This present study used 50 Murrah crossbreed male and female buffaloes between two and six months of age, having been raised on the pasture of a farm in the municipality of Moju, Pará, Brazil. These buffaloes presented no detectable antibody levels against neither BoNTs C and D. They were randomly segregated into five groups of ten animals each. Groups one (Vrec100), two (Vrec200) and three (Vrec400) were vaccinated with non-purified recombinant vaccine against serotypes C and D at concentrations of 100, 200, and 400 µg of recombinant proteins, respectively. Group four (Vcom) was vaccinated with a bivalent commercial toxoid-based (serotypes C and D), while group five (negative control) received 5 mL of sterile saline solution (NaCl 0.9% (w/v)). Immunization was performed subcutaneously on days 0 and 28. Fifty-six days after the first vaccination, blood samples were collected from the jugular vein (Vacutainer^®^, BD, Franklin Lakes, NJ, USA), transported to the laboratory and centrifuged (3000× *g*, 7 min) to obtain serum samples. These were labelled and stored in 2.0 mL microtubes (Eppendorf^®^, São Paulo, SP, Brazil) at −20 °C until further use.

### 4.4. Humoral Immune Response Evaluation

The humoral immune response was evaluated by the serum neutralization bioassay in mice as described by the Brazilian Ministry of Agriculture, Livestock and Food Supply (MAPA) in its normative instruction number 23 (NI 23) [9]. Briefly, sera dilutions were mixed with standard toxins at 37 °C for 60 min, before 0.2 mL of each dilution was inoculated intravenously in two Swiss Webster mice weighing between 18 and 22 g. The animals were observed every 24 h to determine if they were alive or dead for a total period of 72 h. Retro-titration with standard anti-toxins C (5 IU/mL) and D (2 IU/mL) was performed to check the standardization of the toxins.

### 4.5. Statistical Analysis

Minitab^®^ 17.1.0 was used to perform Analysis of variance (ANOVA) and Tukey’s test to identify significant differences in the mean titers of antibodies between groups. A regression analysis was conducted for the concentration of recombinant proteins and titers against BoNTs C and D.

## Figures and Tables

**Table 1:** Neutralizing antibody titers, mean titers, and seroconversion rate against botulinum neurotoxins (BoNTs) serotypes C and D of buffaloes vaccinated with two doses of commercial toxoid (Vcom) and different concentrations of the recombinant vaccine (Vrec). This was tested by the serum neutralization bioassay in mice.

Vaccine	Vrec100		Vrec200		Vrec400		Vcom	
Repetition	C	D	C	D	C	D	C	D
1	0	0	5	4	10	15	5	3
2	5	2	5	2	10	15	5	2
3	5	2	8	8	12	15	5	0
4	0	0	6	8	12	15	6	5
5	0	0	6	2	12	15	5	0
6	5	4	6	4	12	15	6	4
7	0	2	5	10	10	10	5	2
8	0	2	6	8	10	10	0	5
9	5	2	8	8	10	12	5	2
10	5	2	6	8	12	15	5	2
Mean titers	2.5	1.6	6.1	6.2	11.0	13.7	4.7	2.5
Seroconversion rate *	50%	70%	100%	100%	100%	100%	90%	80%

C—BoNT C antitoxin (IU/mL); D—BoNT D antitoxin (IU/mL); * with consideration of the minimum antibodies titers required by NI 23 MAPA.

**Table 2:** Mean titers of immunological responses to Vrec100, Vrec200, Vrec400, and Vcom against botulinum neurotoxins (BoNTs) serotypes C and D, compared by Tukey’s test (*p* < 0.001).

Vaccine	BoNT Mean Titer	
	C	D
Vrec400	11.0 ^a^	13.7 ^A^
Vrec200	6.1 ^b^	6.2 ^B^
Vcom	4.7 ^b,c^	2.5 ^B,C^
Vrec100	2.5 ^c^	1.6 ^C^

Letters were used to point out means titers that were statistically equal or different. Small letters (a–c) were used to compare BoNT C mean titers and capital letters (A–C) were used to compare BoNT D mean titers. Means that do not share a letter are statistically different.
